# Peptidoglycan from Fermentation By-Product Triggers Defense Responses in Grapevine

**DOI:** 10.1371/journal.pone.0113340

**Published:** 2014-11-26

**Authors:** Yang Chen, Taito Takeda, Yoshinao Aoki, Keiko Fujita, Shunji Suzuki, Daisuke Igarashi

**Affiliations:** 1 Institute for Innovation, Ajinomoto Co., Inc., Kawasaki, Kanagawa, Japan; 2 Institute of Enology and Viticulture, University of Yamanashi, Kofu, Yamanashi, Japan; 3 Faculty of Life and Environmental Sciences, Prefectural University of Hiroshima, Shobara, Hiroshima, Japan; Indiana University, United States of America

## Abstract

Plants are constantly under attack from a variety of microorganisms, and rely on a series of complex detection and response systems to protect themselves from infection. Here, we found that a by-product of glutamate fermentation triggered defense responses in grapevine, increasing the expression of defense response genes in cultured cells, foliar chitinase activity, and resistance to infection by downy mildew in leaf explants. To identify the molecule that triggered this innate immunity, we fractionated and purified candidates extracted from *Corynebacterium glutamicum*, a bacterium used in the production of amino acids by fermentation. Using hydrolysis by lysozyme, a silkworm larva plasma detection system, and gel filtration analysis, we identified peptidoglycan as inducing the defense responses. Peptidoglycans of *Escherichia coli*, *Bacillus subtilis*, and *Staphylococcus aureus* also generated similar defensive responses.

## Introduction

Innate immunity is a plant's first line of defense against invasion by microorganisms. The intrinsic immune system helps plants to sense pathogens and rapidly generate defense responses. Without it, plants would not survive. Pathogen- or microbe-associated molecular pattern (PAMP/MAMP)-triggered immunity defends against the attack of plant cells by microbes [1]. Defense is initiated by the recognition of “elicitor molecules” derived from the microbes. Several elicitor molecules are known, and their corresponding receptors have been identified in several plant species. The perception of flagellin by FLS2, a membrane-located receptor-like kinase, induces the innate immune response in *Arabidopsis thaliana* [2]. EF-Tu, one of prokaryotic elongation factors, is recognized by the receptor EFR, which induces the defense response in *A. thaliana* [3], [4]. Chitin, a common fungal molecule, is perceived by the cell-surface receptor CEBiP, which triggers innate immunity in *A. thaliana* [5]–[7]. The bacterial cell wall components lipopolysaccharide (LPS) [8], [9] and peptidoglycan [10] also induce the defense response in plants.

Peptidoglycan is an essential component specific to bacteria. It is a polymer composed of alternating units of N-acetylglucosamine (GlcNAc) and N-acetylmuramic acid (MurNAc) cross-linked via short peptides. This structure allows it to reinforce bacterial walls. As a component of potential pathogens, it is an excellent target for the mammalian innate immune system [11]. It can also trigger the innate immune response in plants. As a PAMP/MAMP, peptidoglycan triggers the defense response in *A. thaliana* [10]. The plasma membrane lysine-motif domain proteins LYM1 and LYM3 were identified as the receptors that recognize peptidoglycan, and LysM receptor kinase CERK1 mediates the signals that switch on the innate immune response in *A. thaliana* [12]. Peptidoglycan also triggers innate immunity in tobacco [13], tomato [14], and rice [15].

The by-products of microbial fermentation in the food industry are widely used as fertilizers and as animal feed, as they contain plenty of nutrients such as amino acids, minerals, and nitrogen compounds. We previously showed that glutamate fermentation by-product (GFB) significantly enhanced the resistance of *A. thaliana* leaves to infection by bacterial pathogens via the activation of defense response signaling and the induction of hydrogen peroxide production [16]. However, the components that trigger this response are yet to be identified. Here, we investigated the defense response of grapevine. GFB triggered a substantial defense response, increasing the expression of defense response genes, foliar chitinase activity, and resistance to infection by downy mildew. By fractionating and testing candidate components, we identified peptidoglycan as the trigger.

## Materials and Methods

### Plant materials

Cultured grape cells were obtained by culturing shoot tips (1–2 cm long) that were collected from *Vitis vinifera* cv. Koshu, since this cultivar is suitable for liquid culture. The tips were surface-sterilized in 70% ethanol for 5 min and then in 10% commercial bleach for 10 min. After washing three times in sterile water, tips containing the meristematic dome and a few leaf primordia were excised in an air-flow cabinet and placed on the surface of half-strength Murashige and Skoog medium containing 1 mg/mL 6-benzylaminopurine, 3% sucrose, and 0.6% agar. Callus induced from the shoot tips was selected and transferred to GB medium, which contained Gamborg's B5 medium salt mixture (Wako, Osaka, Japan), 20 mg/L thiamine hydrochloride, 2 mg/L nicotinic acid, 2 mg/L pyridoxine hydrochloride, 200 mg/L myo-inositol, 58 mM sucrose, 0.54 µM 1-naphthaleneacetic acid, 0.93 µM kinetin, and 0.8% agar. Calluses were grown at 27°C in the dark on medium changed every month. Cell suspension cultures were established by growing callus in a 100-mL flask with 30 mL of GB medium without agar. The cell suspension cultures with small groups of aggregated cells (YU-1) were maintained at 27°C on an orbital shaker (110 rpm) in the dark and subcultured every week with an inoculum dilution of 1/8 (v/v inoculum/fresh medium).

*Vitis vinifera* cv. Chardonnay is a suitable cultivar to grow under experimental conditions, and foliar chitinase activity is easy to be monitored from this cultivar. *Vitis vinifera* cv. Neo Muscat is a suitable cultivar to evaluate disease infection, since it is sensitive to the infection of downy mildew. All the plants were cultured at a photosynthetically active radiation of 100 µmol/m^2^ s under a photoperiod of 16∶8 h L∶D at 23°C unless otherwise indicated.

### Glutamate fermentation by-product

A glutamate fermentation by-product (GFB) manufactured by Ajinomoto Inc.(Tokyo, Japan), was produced by purifying crude glutamate extract obtained from successive filtrations and salting out glutamate [17], [18]. Main components of the GFB are L-glutamate, (NH_4_)_2_SO_4_, NaCl and microbial components.

### Preparation of crude extracts of *Corynebacterium glutamicum*

The fermentation bacteria *Corynebacterium glutamicum* (formerly *Brevibacterium lactofermentum* 2256; ATCC 13869) were grown under general growth conditions [19]. Cells were collected by centrifugation (13 000×*g* for 10 min at 4°C) and washed with 10 mM NaCl solution. The cells were resuspended in 10 mM NaCl solution and homogenized by sonication. Homogenized cell lysates were then heat-denatured at 80°C for 15 min. The denatured cell lysates were centrifuged and resuspended in 10 mM NaCl solution then precipitated with 60% saturated (NH_4_)_2_SO_4_ solution. The precipitated fractions were resusupended in 10 mM NaCl solution and applied to Amicon Ultra centrifugal filters (Millipore, Billerica, MA, USA), and the filtered fractions with a molecular weight (MW) of >50 kDa were collected. These fractions were precipitated with 0.2% (v/v) formic acid and collected by centrifugation and then resuspended in methanol containing 0.5% (v/v) ammonia saturated solution. After evaporation (20°C, 933 Pa) of the methanol and ammonium hydroxide, the dry materials were dissolved in 50 mM Tris·HCl (pH 8.0) buffer to serve as the crude extract. The protein concentration of the crude extract was quantified by Quick Start Bradford protein assay. (Bio-Rad Laboratories, Hercules, CA, USA).

### Purification of active components from *C. glutamicum* crude extract

The crude extract of *C. glutamicum* was filtered through a 0.45-µM membrane filter (Millipore) and loaded on a HiTrap DEAE FF anion exchange column (5 mL, GE Healthcare, Buckinghamshire, UK). Fractions eluted from the column with a linear NaCl gradient of 0–0.5 M in 50 mM Tris·HCl buffer (pH 8.0) were used in defense gene expression assays as described in the next section. Fractions that induced high expression were combined and concentrated, and were loaded on a HiTrap phenyl FF hydrophobic interaction column (5 mL, GE Healthcare). Fractions eluted from the column with a decreasing linear (NH4)_2_SO_4_ gradient of 1–0 M in 50 mM Tris·HCl buffer (pH 8.0) were again used in defense gene expression assays. For gel filtration assay, the concentrated fractions from HiTrap DEAE FF anion exchange column were loaded on a Superdex-200 FF 10/300 GL gel filtration column (GE Healthcare). Fractions eluted from the column with 50 mM phosphate buffer (pH 7.0) containing 0.15 M NaCl were again used in defense gene expression assays. To estimate the MW of candidates that triggered the defense response, we used dextran 2000 (GE Healthcare) as a molecular marker.

### Real-time PCR analysis of defense response genes

Grapevine cells were cultured in 24-well-plates with 0.5 ml Murashige and Skoog medium and the culture was performed at 27°C by continuous shaking. After 16 h culture, the cells were treated with GFB, bacterial extract (2256Ex) or water (mock treatment). After another 5 h, the cells were collected, frozen, and ground into a fine powder in an MM 300 mixer (Qiagen, Chatsworth, CA, USA). Total RNA was extracted with a total RNA extraction reagent (RNAiso Plus; Takara Bio, Otsu, Japan) according to the manufacturer's instructions. cDNA was generated from 0.5 µg of total RNA with a ReverTra Ace qPCR RT Master Mix reagent kit (Toyobo, Osaka, Japan) according to the manufacturer's instructions. Quantitative real-time PCR was performed on an ABI 7500 Fast Real-time PCR System with ABI Fast SYBR Green Master Mix (Life Technologies, Carlsbad, CA, USA). The amplification reaction was performed by two-step PCR in 40 cycles of denaturation at 95°C for 3 s and extension and detection at 60°C for 30 s. The relative abundance of the defense response gene transcripts (stilbene synthase, *STS*; an acidic class IV chitinase, *CHIT-4c*; and β1,3-glucanase, *PR2*) was estimated by normalization against endogenous *V. vinifera β-Actin* (*Vv-Actin*). The primers for each gene were as follows:

*Vv-Actin*-F, 5′-CAAGAGCTGGAAACTGCAAAGA-3′

*Vv-Actin*-R, 5′-AATGAGAGATGGCTGGAAGAGG-3′

*Vv-STS*-F, 5′-AGGAAGCAGCATTGAAGGCTC-3′

*Vv-STS*-R, 5′-TGCACCAGGCATTTCTACACC-3′

*Vv-CHIT4c*-F, 5′-AGATCGCAGCCTTCTTTGGA-3′

*Vv-CHIT4c*-R, 5′-TAATCCTTCCCGGACACACA-3′

*Vv-PR2*-F, 5′-TGGTGCTTCTTCTCGGGTTT-3′

*Vv-PR2*-R, 5′-CCACTTGTGATGCTGGTGGT-3′.

### Assay for chitinase activity

**Figure 1:**
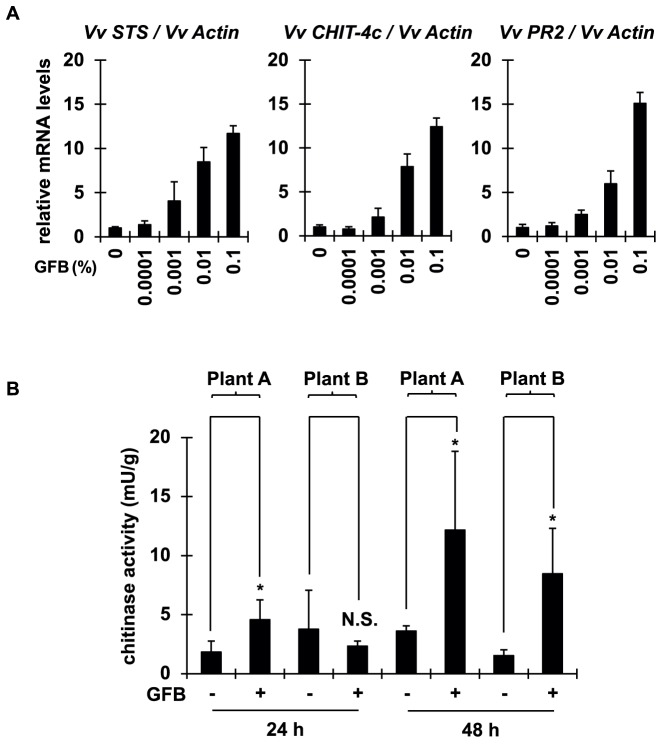
Glutamate fermentation by-product (GFB) elevated expression of defense response genes and foliar chitinase activity in grapevine. (A) Cultured grape cells were treated with GFB at indicated concentrations. Expression of defense response genes *STS*, *CHIT-4c*, and *PR2* was evaluated by real-time PCR at 5 h. (B) Grape leaves from two Chardonnay individual trees (plant A and plant B) were sprayed with 2% (w/v) GFB, and total protein was extracted after 24 or 48 h. Foliar chitinase activity was assayed. All data are means ± S.D. of three biological replicates. *P<0.05, relative to mock treatment; N.S., not significant (Student's t-test).

**Figure 2:**
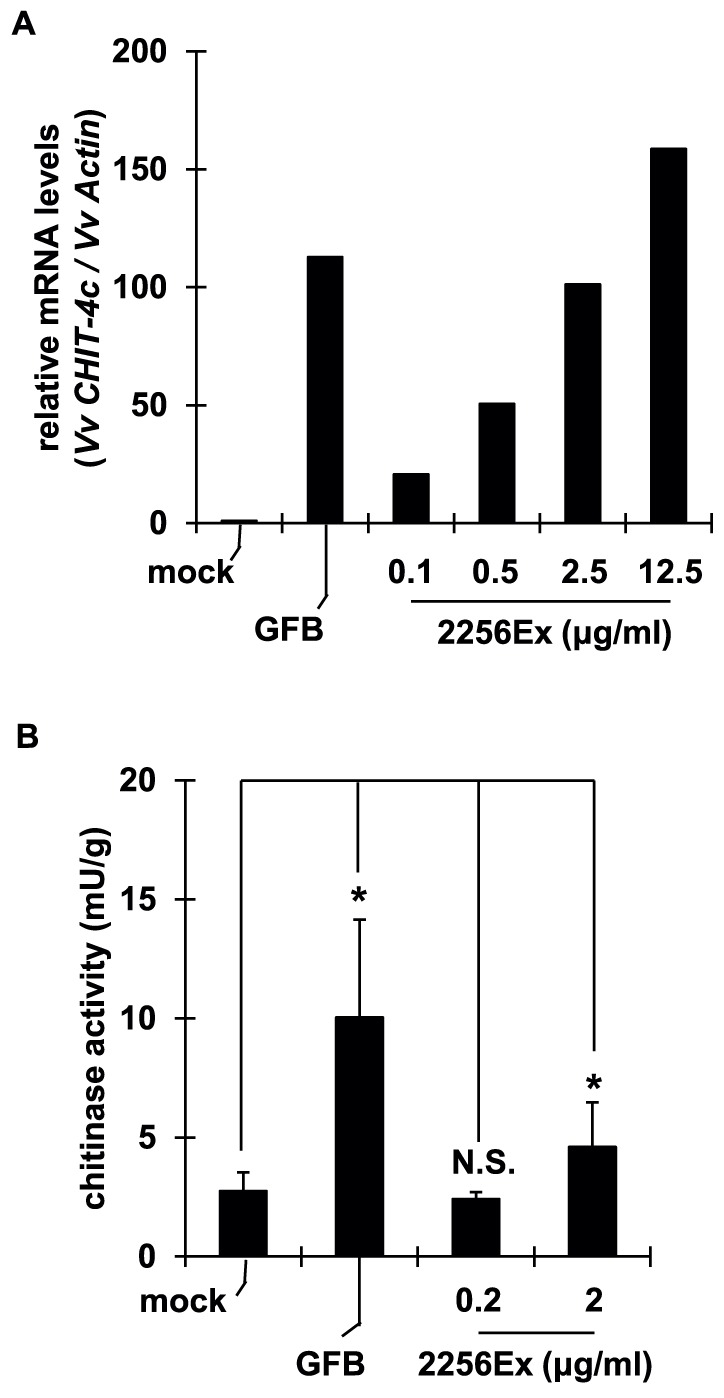
Treatment with extracts of *C. glutamicum* upregulated defense response genes and chitinase activity. (A) Cultured grape cells were treated with crude extracts of *C. glutamicum* (2256Ex) at indicated concentrations. Expression of defense response gene *CHIT-4c* was measured at 5 h. (B) Grape leaves were treated with 2256Ex at indicated concentrations. Foliar chitinase activity was assayed at 48 h. Data are means ± S.D. of three biological replicates. *P<0.05, relative to mock treatment; N.S., not significant (Student's *t*-test).

Fully expanded leaves of Chardonnay were detached from plants and laid in 10-cm Petri dishes with moistened filter papers. The leaves were sprayed with 2% (w/v) GFB or *C. glutamicum* extract and incubated in the light at 23°C for 24 or 48 h as indicated in Fig. 1B and Fig. 2B. The leaves were then frozen and ground into a fine powder in an MM 300 mixer (Qiagen). Total protein was extracted from the powder with a P-PER plant protein extraction kit (Thermo, Rockford, IL, USA) according to the manufacturer's manual. The protein extracts were then diluted with reaction buffer (0.1 M Na_2_HPO_4_/NaH_2_PO_4_, pH 6.0, with 10 mM dithiothreitol), and total protein was quantified with a BCA Protein Assay kit (Thermo).

Chitinase activity was measured using 40 mM 4-methylumbelliferyl β-d-N,N′,N″-triacetylchitotrioside (4-MU-(GlcNAc)_3_; Sigma, St. Louis, MO, USA) as a substrate. Leaf protein extract (50 µL, 0.5 mg/mL) was mixed with 50 µL of substrate solution and incubated at 37°C for 90 min. The reaction was terminated by the addition of 100 µL of 1 M Gly-NaOH (pH 10.2). The hydrolyzed 4-MU was monitored by spectrofluorometer (SpectraMax M2; Molecular Devices, Sunnyvale, CA, USA) with excitation at 360 nm and emission at 450 nm. One unit (U) of chitinase activity was defined as the amount of enzyme that catalyzed the conversion of 1 µmol of 4-MU-(GlcNAc) per minute under the assay conditions.

### *In vivo* bioassay for infection by grape downy mildew

The third to fifth leaves, counted from the shoot tip, were collected from *V. vinifera* cv. Neo Muscat seedlings. Disks (15 mm diameter) were punched out from the leaves and placed upside down on 0.5% agar in Petri dishes. The leaf disks were sprayed with 5 mL of 2% (w/v) GFB, 2 mg/mL 2256Ex, 3.3 mg/L mandipropamid,MDPP (Revus flowable, 23.3% mandipropamid stock solution, Syngenta Japan, Tokyo, Japan) served as positive control, or water (each containing 0.1% Approach BI spreader [Maruwa Biochemical, Tokyo, Japan] as a fixer) from an atomizer. The surface of the leaf disks was dried in a flow cabinet for 24 h.

Downy mildew–infected grape leaves were placed in a Petri dish with a moisture filter paper. After incubation for 3 days, spores of *Plasmopara viticola* were washed off with sterile water and adjusted to a concentration of 50 000 spores/mL. Each pretreated leaf disk was then inoculated with 20 µL of the spore suspension from a pipette. The leaf disks were incubated in the light at 21°C in a plastic box containing a moistened paper towel to achieve ∼100% humidity.

Disease symptoms of downy mildew on each leaf disk were assessed 7 days after inoculation and scored on a disease index of 0 to 4 as follows: 0, no symptoms; 1, white growth on up to 1/6 of the disk; 2, white growth on up to 1/3 of the disk; 3, white growth on up to 1/2 of the disk; 4, white growth on more than 1/2 of the disk.

### Lysozyme treatment and Silkworm larva plasma (SLP) detection system

Presence of peptidoglycan in the purified fraction from AEC and HIC was validated by lysozyme treatment. GFB or HIC fraction was pre-incubated with chicken egg derived lysozyme (Sigma) at 37°C for 3 h, and subsequently subjected to YU-1 culture suspension. After 5 h incubation, transcription levels of defense response genes were evaluated. We also assayed peptidoglycan in the GFB or crude extract of *C. glutamicum* by using a silkworm larva plasma (SLP) reagent set (Wako, Osaka, Japan). Peptidoglycan promotes the prophenoloxidase cascade in SLP [20]. Diluted solutions of purified peptidoglycan standards obtained from *Micrococcus luteus* (Wako) or samples were mixed with the substrates, and the mixtures were incubated at 30°C to promote the reaction. Kinetics of the absorption changes in the reaction mixture at 650 nm was monitored as described in the manufacturer's manuals, and the time at which absorption reached 0.3 (Fig. S1A) was plotted against the concentration of standard to create a standard curve (Fig. S1B). The peptidoglycan concentration of samples was calculated from this curve.

## Results

### GFB-triggered defense response in grapevine

To investigate the defense-response-inducing effect of GFB, we serially diluted the GFB to treat cultured grape cells and evaluated the expression of typical defense response genes, namely *STS* (stilbene synthase; [21]) *CHIT-4c* (an acidic class IV chitinase; [22]) and *PR2* (β1,3-glucanase; [23]). Treatment with GFB elevated the expression of all genes dose-dependently (Fig. 1A). Since the elevation tendency of all defense genes expression is similar, we chose *CHIT-4c* as a representative gene to evaluate defense response in the following experiments. To investigate the defense response triggered by GFB at tissue level, the foliar chitinase activity was measured. We sprayed 2% (w/v) GFB solution on the leaves of two Chardonnay plants. After 24 or 48 h incubation, we extracted the total protein and assayed chitinase activity against 4-MU-(GlcNAc)_3_ substrate. No significant change was evident by 24 h, but chitinase activity was greatly elevated by 48 h (Fig. 1B). Both sets of results show that GFB triggers the defense response at both cellular and tissue levels in grapevine.

### A component of *C. glutamicum* triggers innate immunity in grapevine

Components of pathogens can reinforce innate immunity in plant species. We investigated whether bacterial components in GFB triggered the defense response. We prepared crude extracts of *C. glutamicum* 2256 (2256Ex), which is similar to the bacteria used in glutamate fermentation. The extracts were serially diluted and applied to cultured cells or fresh leaves. We monitored transcript levels of defense response genes and foliar chitinase activity. Treatment with 2256Ex elevated gene expression dose-dependently (Fig. 2A). Treatment with 2 mg/mL of 2256Ex significantly increased foliar chitinase activity (Fig. 2B). However, treatment with other major components of GFB, namely (NH_4_)_2_SO_4_, NaCl, and monosodium glutamate, did not trigger an obvious defense response (Fig. S2). Thus, a bacterial component of *C. glutamicum* in GFB triggered the defense response.

### Defense response induced by GFB enhanced resistance to infection by downy mildew

**Figure 3:**
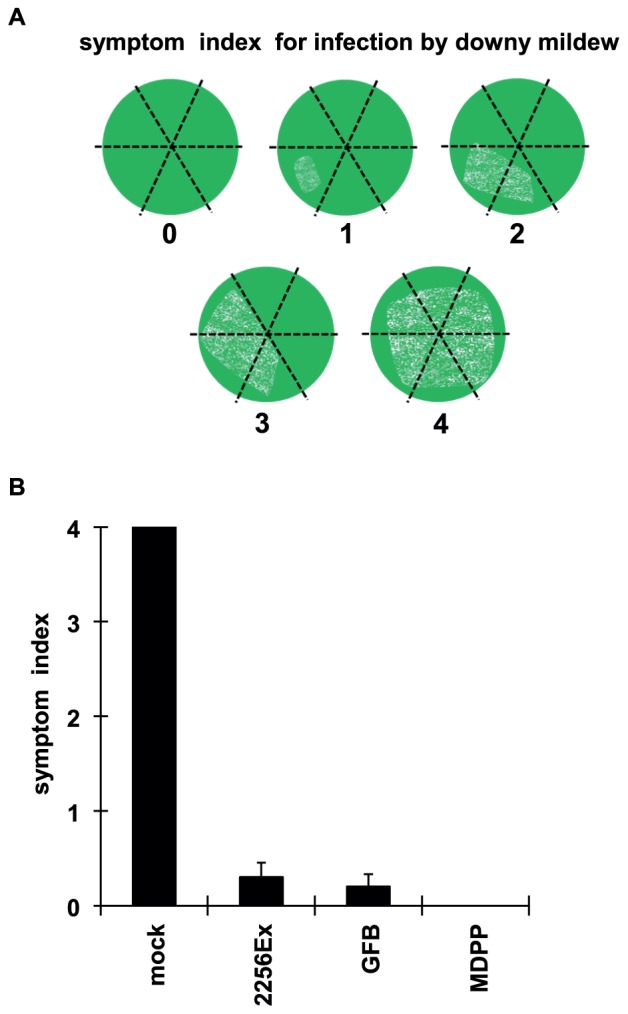
Glutamate fermentation by-product (GFB)-triggered defense response enhanced resistance to infection by downy mildew. (A) Leaf disks of Neo Muscat were treated with 2% GFB, 2 mg/mL 2256Ex, 3.3 mg/L mandipropamid (MDPP), or water (mock), and inoculated with *P. viticola*. After 7 days, infection was scored as: 0, no infection; 1, <1/6 of the leaf disk; 2, >1/6 but <1/3 of the leaf disk; 3, >1/3 but <1/2 of the leaf disk; 4, >1/2 of the leaf disk. (B) Evaluated symptoms. Data are means ± S.D. of 10 biological replicates.

To investigate whether the defense response triggered by GFB or crude extracts of *C. glutamicum* could enhance resistance to downy mildew, we performed an *in vivo* assay. Leaf disks pretreated with GFB or crude extracts of *C. glutamicum* were inoculated with *P. viticola* as indicated in materials and methods. After 7 days, disease symptoms were assessed to evaluate infection by downy mildew (Fig. 3A). Leaf disks pretreated only with water showed severe disease symptoms, and treatment with a fungicide named mandipropamid completely abolished the disease symptoms. Whereas those pretreated with GFB or crude extracts showed limited symptoms (Fig. 3B). Thus, a bacterial component from the GFB triggered defense response and apparently enhanced the innate immunity in grapevine, limiting infection by the pathogen.

### Peptidoglycan triggered innate immunity in grapevine

**Figure 4:**
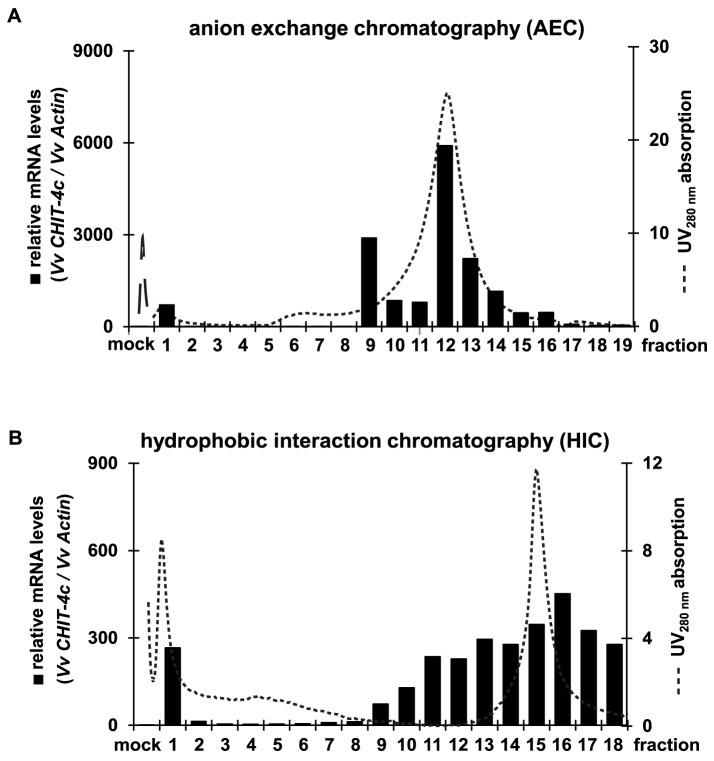
Purification of defense-triggering molecules. (A) 2256Ex was separated by anion exchange chromatography (AEC). Cultured grape cells were treated with each fraction or water (mock) and expression of defense gene *CHIT-4c* was measured. (B) AEC fraction 12 was separated by hydrophobic interaction chromatography. Cultured cells were treated with each fraction and expression of defense gene *CHIT-4c* was measured. Left *y*-axes show relative expression of defense response gene. Right *y*-axes show absorption at 280 nm.

The observation that 2256Ex triggered defense response, that implies the presence of defense response inducing molecules in the bacterial components. Thus, we performed anion exchange chromatography (AEC) and hydrophobic interaction chromatography (HIC) to purify the molecules that induced the defense response. First we simply purified the 2256Ex by passing through centrifugal filter units. When fractions of 2256Ex with a MW of <50 kDa were removed, most of the ability to trigger the defense response was retained (Fig. S3). Filtered samples were precipitated in 0.2% (v/v) formic acid and dissolved in 0.5% (v/v) ammonia water, and passed through a HiTrap-DEAE FF column to perform AEC. The purified fractions were applied to cultured cells to evaluate the expression of defense response gene (Fig. 4A). Fractions with activity were collected and then passed through a HiTrap-Phenyl FF column to perform HIC. A hydrophilic fraction (HIC-fraction 1) and several hydrophobic fractions (11–18) showed high defense-response-inducing activity (Fig. 4B). Finally, we collected HIC-fractions 1 and 16 to identify candidates.

**Figure 5:**
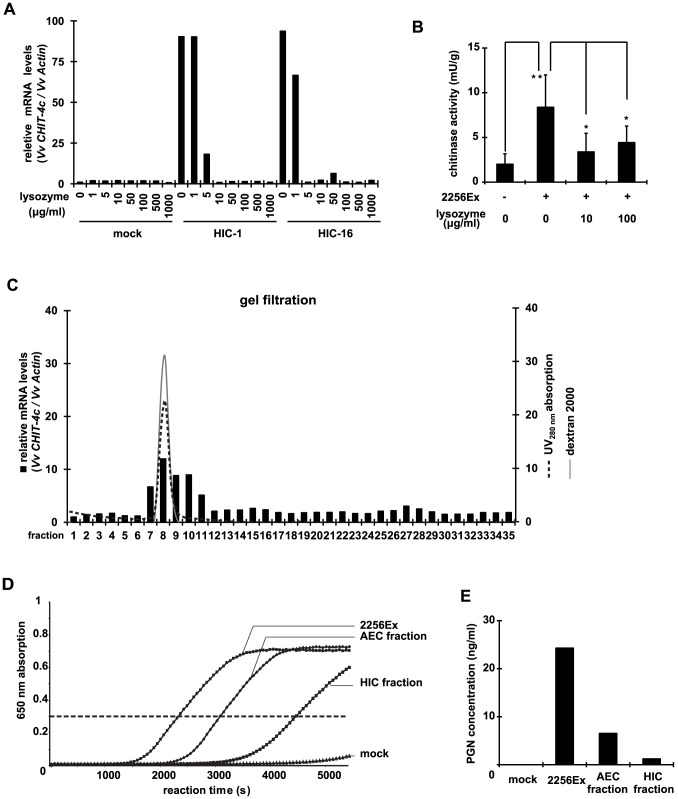
Peptidoglycan triggers defense response in grapevine. (A) Fractions 1 and 16 from HIC (Fig. 4B) and water (mock) were treated with lysozyme at indicated concentrations and applied to cultured grape cells. Expression of defense response gene *CHIT-4c* was evaluated at 5 h. (B) Foliar chitinase activity treated with 2256Ex that was hydrolyzed by lysozyme at indicated concentrations. Data are means ± S.D. of five biological replicates. *P<0.05; **P<0.01 (Student's t-test). (C) Fraction 12 from AEC (Fig. 4A) was separated by gel filtration chromatography. To estimate the MW of candidate molecules that triggered defense response genes, dextran 2000 was used as a molecular marker. (D) Peptidoglycans in 2256Ex, AEC fraction, and HIC fraction were quantified by SLP detection system. Test fractions were mixed with substrate and incubated at 30°C, and the Kinetics of the absorption changes in the reaction mixture at 650 nm was monitored. (E) According to the reaction time at which absorption reacted 0.3, concentration of peptidoglycans (PGN) in D was calculated.

Since PAMPs/MAMPs can trigger innate immunity in plants, we postulated that PAMPs/MAMPs of *C. glutamicum* promoted the defense response. Among PAMPs/MAMPs, peptidoglycan, the most abundant component of the cell wall of *C. glutamicum*, is likely. To test this possibility, we performed enzyme hydrolysis assay using lysozyme. Lysozyme is widely distributed in the secretions of vertebrates, and prevents infection by pathogenic microorganisms. It degrades the bacterial cell wall via hydrolysis of the 1,4-beta-linkages between MurNAc and GlcNAc structures in peptidoglycan. We pre-incubated HIC-fractions 1 and 16 with lysozyme, and evaluated the effects of these treated fractions on triggering of the defense response. Pre-incubation with high concentrations of lysozyme dramatically reduced the expression of defense response genes dose dependently (Fig. 5A) and downregulated foliar chitinase activity (Fig. 5B), which 2256Ex had upregulated.

Gel filtration analysis revealed that the main component of peptidoglycan that induced the defense response had a MW of approximately 2000 kDa (Fig. 5C). The SLP detection system confirmed the presence of peptidoglycan in the same 2256Ex fractions (Fig. 5D, E). All results show that a high-MW peptidoglycan in *C. glutamicum* crude extract triggered the defense response. However, chitosan and flg22—two other PAMPs/MAMPs—did not induce expression (Fig. S4).

### Peptidoglycans from other fermentation by-products also induced defense response

**Figure 6:**
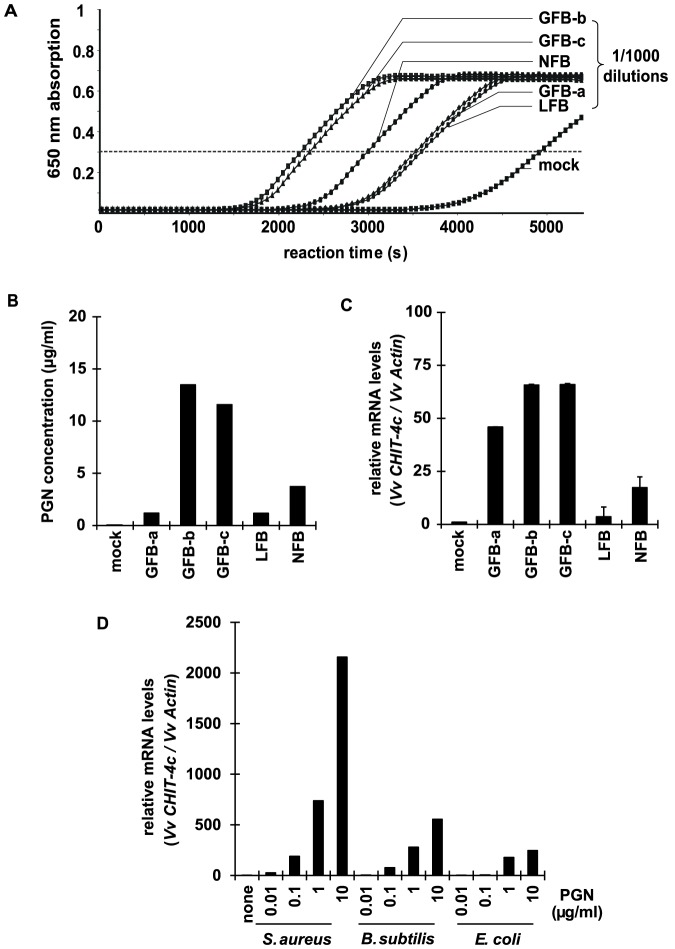
Defense response in cultured grape cells treated with peptidoglycans from fermentation by-products of *C. glutamicum* (GFB-a, GFB-b, GFB-c), *E. coli* (LFB), *Bacillus subtilis* (NFB), and water (mock). (A) Quantification of peptidoglycans by SLP detection system. (B) Calculated concentrations of peptidoglycans (PGN) in these by-products. (C) Expression of defense response gene *CHIT-4c* in treated grape cells. (D) Expression of *CHIT-4c* in grape cells treated with peptidoglycans (PGN) purified from *S. aureus*, *E. coli*, and *B. subtilis*.

We investigated the effects of by-products from other fermentation processes on the defense response. By-products from fermentation by *C. glutamicum* (GFB-a, GFB-b, GFB-c), *Bacillus subtilis* (NFB), and *Escherichia coli* (LFB) were applied to cultured grape cells. The SLP detection system revealed the presence of peptidoglycan in all of these by-products (Fig. 6A, B), and all by-products increased the expression of defense response gene (Fig. 6C). We also applied purified peptidoglycans from *E. coli*, *B. subtilis* and *S. aureus* to cultured cells to evaluate their effects on the defense response. Although there are differences between Lys-type (*S. aureus*) and *meso*-diaminopimelic acid (mDAP)-type (*E. coli*, *B. subtilis*) peptidoglycans [24], all of these peptidoglycans increased the expression of defense response genes dose-dependently (Fig. 6D). These results imply the essential role of peptidoglycan in the recognition of pathogenic microbes by grapevine cells, which triggers defense response.

## Discussion

Our previous study demonstrated that GFB strengthened the defense of *A. thaliana* leaves against *Pseudomonas syringae* pv. *maculicola*, and that a bacterial component was a likely factor [16]. Here, we clarified that the component is peptidoglycan, the main component of the bacteria cell wall. PAMP/MAMPs from pathogenic and non-pathogenic microbes are essential elicitors of the plant defense response, and include flagellin [25], EF-Tu [3], LPS [8], [9], and peptidoglycan [10]. Both *A. thaliana* and tomato are highly sensitive to flagellin [26]. Recognition of the EF-Tu N-terminus elicits innate immunity in members of the Brassicaceae [4], but perception of the middle part of EF-Tu elicits it in rice [27]. Promotion of the defense response by LPS has been studied mostly in dicots and in some monocots such as rice [9], [28]. The perception of peptidoglycan was first found in *A. thaliana* [10], followed by tomato [14], tobacco [13], and rice [15]. Here, we added grapevine, extending the evidence to woody species.

Recognition of PAMP/MAMPs by the corresponding receptors on the plant cell surface is a key factor in initiating the early immune response in plants. The plasma membrane Lys-motif domain proteins LYM1 and LYM3 were identified as peptidoglycan receptors, initiating the defense response [12]. Recognition by the cell surface receptors and thus detection by the plants possibly depends on the structure of the peptidoglycan. Treatment with lysozyme significantly reduced the defense response (Fig. 5A, B). This observation implies the possible roles of glycan structure in recognition by the receptors. High-MW peptidoglycans elicited the defense response most strongly (Fig. 5C). Structural investigations suggested that peptidoglycan with a high MW induced the synthesis of antibacterial proteins, in which the minimum structure required is a peptidoglycan with two repeating muramyl peptide units [29]. Although all of the peptidoglycans from *E. coli*, *B. subtilis*, and *S. aureus* triggered a response, the levels of the responses differed (Fig. 6D). Similarly, peptidoglycan from *Xanthomonas campestris* more strongly induced the innate immunity in *A. thaliana* than that from *Rhizobium radiobacter* [30]. These results imply that minor changes in the peptide parts of peptidoglycans explain different levels of defense response. However, the structural details that influence the perception of peptidoglycan by the receptors are yet to be clarified.

Although we identified the role of peptidoglycan in triggering innate immunity in grapevine, the pathways involved and mechanisms of the defense response are yet to be clarified. Peptidoglycan is the main substance of bacterial cell walls. The cell walls account for most of the total weight in Gram-positive bacteria. It is possible that GFB, the by-product from fermentation by the Gram-positive *C. glutamicum*, contains a high concentration of peptidoglycan. However, other molecules in GFB might also contribute to the immune response, as treatment with lysozyme did not completely abolish the enhanced expression of defense response genes (Fig. S5). Further studies should focus on identifying these other substances.

Our results suggest a possible role for GFB in plant protection, in addition to its nutrient benefits.

## Supporting Information

Figure S1 **Quantification of peptidoglycans by SLP detection system.** (A) Purified peptidoglycan standards from *S. aureus* were diluted as indicated, mixed with substrate, and incubated at 30°C. Kinetics of the absorption changes in the reaction mixture at 650 nm was monitored, and the time when the absorption reached 0.3 was noted. (B) The standard curve was based on the linear relation between the concentration and the recorded time length of each standard.
(EPS)

Figure S2 **Cultured grape cells treated with non-microbial components of GFB showed no significant defense response.** Cells were treated with (NH_4_)_2_SO_4_, NaCl, or monosodium glutamate (MSG) at concentrations equivalent to those in 0.1% (w/v) GFB and water (mock), Expression of defense response gene *CHIT-4c* was evaluated at 5 h.
(EPS)

Figure S3 **Relative expression of defense response gene** ***CHIT-4c*** **in cultured grape cells treated with indicated fractions of** ***C. glutamicum*** **extract (2256Ex).**
(EPS)

Figure S4 **Cultured grape cells treated with 100 µg/mL chitosan or 1 µM flg22 showed no significant defense response at 5 h.**
(EPS)

Figure S5 **Treatment of GFB with lysozyme partially abolished the GFB-enhanced defense response.** Cultured grape cells were incubated with 0.05% (w/v) GFB treated with lysozyme at indicated concentrations. Expression of defense response gene *CHIT-4c* was evaluated at 5 h.
(EPS)
